# A spatial approach for the epidemiology of antibiotic use and resistance in community-based studies: the emergence of urban clusters of *Escherichia coli *quinolone resistance in Sao Paulo, Brasil

**DOI:** 10.1186/1476-072X-10-17

**Published:** 2011-02-28

**Authors:** Carlos RV Kiffer, Eduardo CG Camargo, Silvia E Shimakura, Paulo J Ribeiro, Trevor C Bailey, Antonio CC Pignatari, Antonio MV Monteiro

**Affiliations:** 1 Special Microbiology Laboratory, Federal University of São Paulo (UNIFESP), São Paulo, Brazil; 2 Earth Observation General Coordination, Image Processing Division, National Institute for Space Research, São José dos Campos, São Paulo, Brazil; 3 Statistics and Geoinformation Laboratory, Federal University of Paraná, Curitiba, Paraná, Brazil; 4 Department of Mathematics, University of Exeter, Exeter, Devon, UK; 5 GC-2 Pharmaceutical Research and Development, Llc, São Paulo, Brazil

## Abstract

**Background:**

Population antimicrobial use may influence resistance emergence. Resistance is an ecological phenomenon due to potential transmissibility. We investigated spatial and temporal patterns of ciprofloxacin (CIP) population consumption related to *E. coli *resistance emergence and dissemination in a major Brazilian city. A total of 4,372 urinary tract infection *E. coli *cases, with 723 CIP resistant, were identified in 2002 from two outpatient centres. Cases were address geocoded in a digital map. Raw CIP consumption data was transformed into usage density in DDDs by CIP selling points influence zones determination. A stochastic model coupled with a Geographical Information System was applied for relating resistance and usage density and for detecting city areas of high/low resistance risk.

**Results:**

*E. coli *CIP resistant cluster emergence was detected and significantly related to usage density at a level of 5 to 9 CIP DDDs. There were clustered hot-spots and a significant global spatial variation in the residual resistance risk after allowing for usage density.

**Conclusions:**

There were clustered hot-spots and a significant global spatial variation in the residual resistance risk after allowing for usage density. The usage density of 5-9 CIP DDDs per 1,000 inhabitants within the same influence zone was the resistance triggering level. This level led to *E. coli *resistance clustering, proving that individual resistance emergence and dissemination was affected by antimicrobial population consumption.

## Background

Mortality due to infections represents approximately 85% of all deaths worldwide [1], and community acquired ones are highly prevalent [1,2]. Furthermore, most of the community infections present a risk of resistance acquisition to a first line antimicrobial drug [1]. The increasing burden of antimicrobial resistance associated to the relative drought on the antimicrobial developmental pipeline has urged for strategies to reduce antibiotic consumption and to maximize treatment approaches [3,4]. These strategies, as stated by Lipsitch and Samore [3], "rest on the well-supported idea that the use of antimicrobial agents is a powerful selective force that promotes the emergence of resistant strains". Although infections are ecological phenomena [3,5-7] and require a new analytical paradigm, researches have generally taken a case-by-case approach to understanding the emergence of new infections or of its consequences [6]. Nevertheless, for any infectious disease, the infection or colonization status of a subject affects the acquisition risk of this very same condition by others and this is due to the transmissibility risk [3], or the so-called ecological phenomenon. Based on this, antibiotic usage as a driver of resistance shall be equally affected. Should a person or a population be submitted to an antibiotic usage, other people around who have not received antibiotics may have increased colonization or infection risk of acquiring a resistant organism [3]. Thus, it is reasonable to assume that population antimicrobial usage may be a major driver in determining the emergence of resistance in community. However, a potential population pressure affecting antimicrobial resistance emergence has not been quantitatively demonstrated so far.

The EUREQA Project (*Epidemiologia do Uso e da Resistência de Antibióticos e Quimioterápicos na população*) [8] is a multi-phase spatial and temporal project aimed at correlating population risk factors, mainly antimicrobial usage density, with community bacterial resistance. The present study aimed at establishing quantitative spatial correlations among antimicrobial usage mean density as measured for the entire population and resistance to ciprofloxacin in community urinary tract infections (UTI) caused by *E. coli *isolated from adult women in a major urban environment, São Paulo city, which occupies an area of only 0.02% of the Brazilian Territory with a population of approximately 10.5 million inhabitants.

## Methods

The present study has been submitted and approved by the Ethics Committee of the Federal University of Sao Paulo (UNIFESP, process CEP 0123/05) and it was based on data observation without patient identification.

### Data

The EUREQA database stores two essential informations: i) UTI *E. coli *events by individual address; ii) antimicrobial selling points. *E. coli *events had the following case definition: routine urine culture results positive for *E. coli *(single isolate per culture, with ≥ 100.000 UFC/ml), collected on two large healthcare outpatient facilities, covering a public and a private sector unit. Since *E. coli *occurs most commonly in female patients, age and sex-specific restrictions were adopted and only data from female patients with age higher than 16 years-old were included in this first analysis. In order to detect resistance clusters, cases were categorized in susceptible (antibiogram = sensitive) or resistant (antibiogram = resistant or intermediate) to ciprofloxacin. Ciprofloxacin was the antimicrobial chosen based on its rapid resistance development by *E. coli *and other Gram-negative bacteria [9-12]. The antimicrobials selling points were obtained from a pharmaceutical market auditing company, IMS Health Brazil, covering 98% of the studied area. They were categorized as drugstore, delivery pharmacy, pharmacy chain, hospital, or major distributor. Those categorized as hospitals and major distributors were excluded from the final database, which focused on the community market exclusively. Each selling point contained monthly Defined Daily Doses (DDD) information of the all antimicrobials included in the EUREQA Project (all with ATC code [13]). The DDD by WHO definition [13] was the basic unit chosen for building a population antimicrobial consumption measurement. For the present study, DDDs for ciprofloxacin and other quinolones (nalidixic acid, norfloxacin, levolfoxacin, gatifloxacin, moxifloxacin) were kept in the database. For each selling point, a mean cumulative DDD of a selected antimicrobial (i.e. ciprofloxacin) for the previous three months (DDDA) was calculated. Exceptions included the months of the January and February, which used replicated values, according to the following: January, DDDA_*Jan *_= 3*(DDD_*Jan*_)/3, and February, DDDA_*Fev*_=(1.5*(DDD_*Jan*_) +1.5*(DDD_*Fev*_)/3. The selection of three months window accumulated data was based on time-series studies in hospitals where resistance associated to increased antimicrobial consumption emerged usually no later than this timeframe in gram-negatives [10].

### Address geocoding

**Figure 1:**
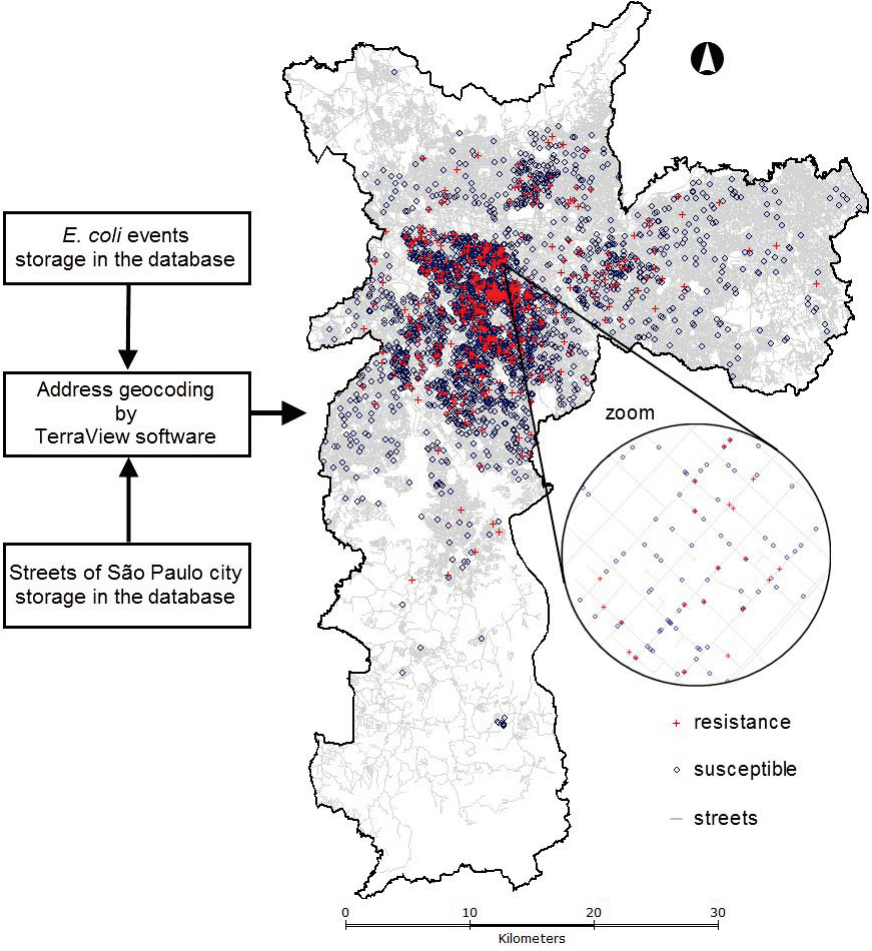
**Address geocoding of the *E. coli *cases (resistant and susceptible) in  São Paulo city, 2002**.

The address geocoding is a procedure to establish the geographical coordinates of the investigated data based on street map storage in the database. Then, *E. coli *events were address geocoded in a digital map (Figure 1) by TerraView [14] software. Similarly, each selling point including ciprofloxacin and other quinolones were address geocoded. The result is similar to that shown in Figure 1. At this point we have mapped the distribution of the *E. coli *events and antimicrobials selling points over the city.

### Selling points influence zone determination

**Figure 2:**
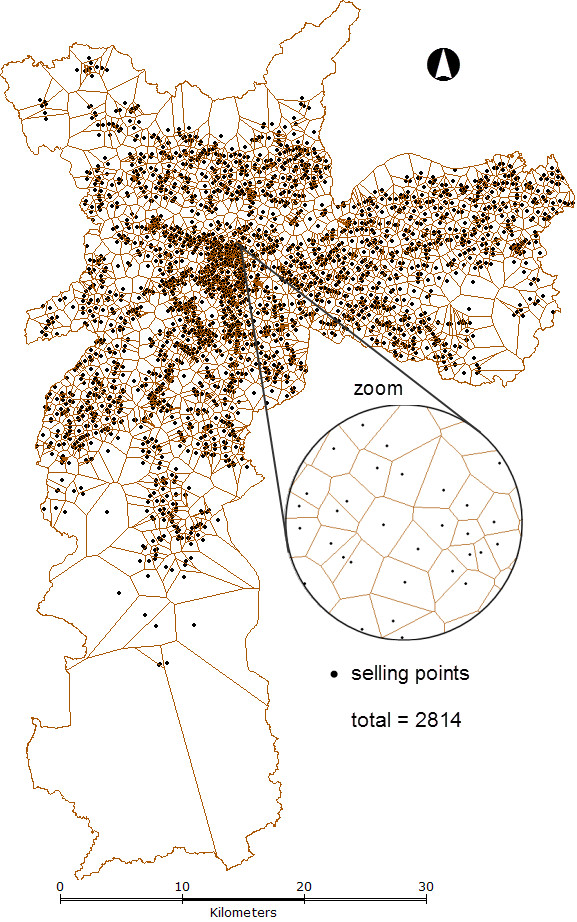
**Selling points influence zones to ciprofloxacin in  São Paulo city, 2002**.

An influence zone was established for each geocoded selling point applying Voronoi Diagrams [15-17]. The result is a polygon map covering the study area (Figure 2), where each polygon represents an influence zone of the respective selling point. Then, all monthly DDDA values associated with a particular selling point was extended to its respective influence zone by TerraView [14] software.

Influence zones were used as the best explanation for consumption pattern, since pharmacy consumption, either by direct buy or delivery, originates from a zone closer to the subject`s residence. This way, a DDDA value initially belonging to a point, was distributed over an area, basically considering that all individuals over that area were submitted to that consumption influence. This procedure was irrespective of individuals actually using or not the antimicrobials measured. It only considered that individuals were under the same consumption influence and, thus, under the same risk for resistance acquisition due to transmissibility or indirect effect [3].

### Antimicrobial consumption density determination

In order to define a population antimicrobial consumption density the antimicrobial usage mean density (D_DDDA) was calculated for each influence zone. Comparison unit was defined as:

$$\text{D}\_{\text{DDDA}}_{k,t}=\frac{{\text{DDDA}}_{k,t}}{\left(\frac{Pop_{k}}{1,000}\right)*30},$$

where DDDA_*k,t *_is the average of the DDD cumulative for three months for *k-th *influence zone and for *t-th *month (*t *= 1,2,..., 12), *Pop_k _/*1,000 is the estimated population by a thousand inhabitants restricted to *k-th *influence zone and *30 *is the number of days in the month.

The population information is in the census tract database. To calculate the D_DDDA_*k,t *_it is necessary to estimate *Pop_k _*the population to each influence zone. It is done in TerraView [14] through an intersection operation between the two maps, influence zone and census tract maps. Then, the population for the *k-th *influence zone was estimated as follows:

$$Pop_{k}=\sum_{i=1}^{n}CTPop_{i}\left(\frac{INTArea_{k,i}}{CTArea_{i}}\right),$$

where *n *is the number of the intersections areas that occurs between the *k-th *influence zone and the census tract map, *i *is the *i-th *census tract, *CTPop_i _*is a population of the *i-th *census tract, *CTArea_i _*is the area of the *i-th *census tract and *INT_Area_k,i _*is the intersection area between of the *k-th *influence zone and the *i-th *census tract.

**Figure 3:**
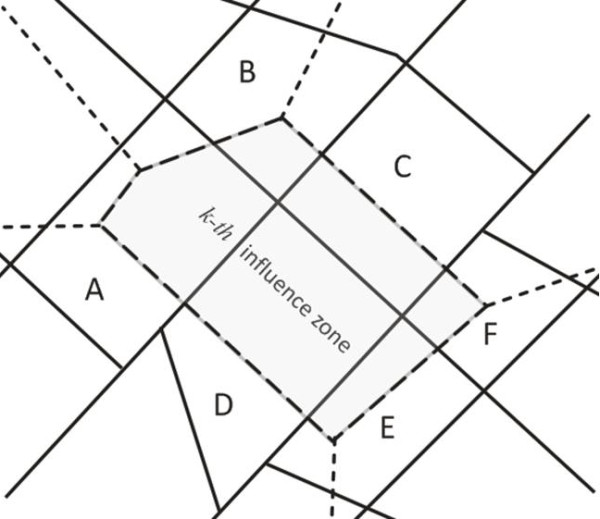
**An example of the population estimation in the *k-th *influence zone**.

To illustrate this operation consider the example of the Figure 3, where the polygons with solid lines represent the census tract (A, B, C, D, E and F) and the polygon with dashed line represents the *k-th *influence zone.

The population in the *k-th *influence zone is equivalent to the weighted sum of the census tract populations A, B, C, D, E and F. The weight is given by the ratio between the intersection area (of the *k-th *influence zone with the *i-th *census tract) and the census tract area. Thus,

$$\begin{array}{l} Pop_{k}=CTPop_{A}\left(\frac{INTArea_{k,A}}{CTArea_{A}}\right)+CTPop_{B}\left(\frac{INTArea_{k,B}}{CTArea_{B}}\right)+CTPop_{C}\left(\frac{INTArea_{k,C}}{CTArea_{C}}\right)\\ \;\;\;\;+\;CTPop_{D}\left(\frac{INTArea_{k,D}}{CTAreaD}\right)+CTPop_{E}\left(\frac{INTArea_{k,E}}{CTArea_{E}}\right)+CTPop_{F}\left(\frac{INTArea_{k,F}}{CTArea_{F}}\right) \end{array}$$

Finally, each resistant or susceptible case contained within an influence zone of the respective usage density, received a value of the respective D_DDDA_*k,t *_according to date of occurrence (closest possible data of the D_DDDA_*k,t *_measurement).

### Generalized additive model: a semi-parametric binary regression approach

To detect resistance clusters in the study area, a spatial Generalized Additive Model (GAM) [18,19] was adopted relating *E. coli *resistance and relevant covariates, spatial and temporal effects. Both the global test for the spatial variation of resistance risk and the identification of areas of high/low risk were performed using the Monte Carlo simulation method proposed by Kelsall and Diggle [20].

### Risk measure

The modeling structure used is based upon a spatial point process [21] under which a risk measure can be defined and estimated continuously over the study region. This procedure firstly defines a risk measure to detect ciprofloxacin resistant clusters in the study area *A *[20]. For this, the susceptible cases are treated as a sample of the population at risk: the ciprofloxacin non resistant (susceptible) residents of São Paulo city.

Consider $\left(s_{\text{1}},s_{\text{2}},...,s_{n_{\text{1}}}\right)$ the locations of the *n*_1 _resistant cases and $\left(s_{n_{\text{1}}+\text{1}},s_{n_{\text{1}}+\text{2}},...,s_{n}\right)$ the locations of the (*n *- *n*_1_)susceptible cases in the study region *A *as observations of two Poisson processes I and II, with intensities λ_1 _(*s*) and λ_2 _(*s*), respectively. A log risk measure at location *s *can be defined as *ρ *(*s*) = log(λ_1 _(*s*)/λ_2 _(*s*)) and the aim is to investigate the spatial variation of *ρ*(*s*) on *A*.

### Risk estimation

A semi-parametric approach to estimating *ρ*(*s*) is used, called the Generalized Additive Model (GAM) [20]. The GAM method allows estimating the spatial risk whilst controlling for potential individual or local environmental factors (such as exposure to D_DDDA, for instance).

Let *y_i _*be an indicator associated to location *s_i _*, such that *y_i _*= 1 if subject *i *is a resistant case or 0 otherwise. Assume that *y_i_*(*i=*1,...,*n*) are realizations of independent Bernoulli random variables *Y_i _*~ *Bernoulli*(*p *(*s*)), where

$$p\left(s\right)=\frac{q_{\text{1}}\lambda_{\text{1}}\left(s\right)}{q_{\text{1}}\lambda_{\text{1}}\left(s\right)+q_{\text{2}}\lambda_{\text{2}}\left(s\right)},$$

and *q*_1 _and *q*_2 _are the sampling proportion of resistant and susceptible cases in relation to the existing total in the population. This allows for the possibility of under-registration under the assumption of missing at random in space. Under this assumption, it follows that

$$log\left\{\frac{p\left(s\right)}{\text{1}-p\left(s\right)}\right\}=\rho \left(s\right)+c,$$

Where *c *= *log*(*q*_1_/*q*_2_), so *c *is simply an additive constant, therefore it does not modify the overall characteristics of the spatial distribution of the risk over the region. Hence, one can obtain estimates of *ρ*(*s*), apart from an additive constant, with a model fitted to a binary outcome. For easy of interpretation the logarithmic scale is used with a base 2, as in this scale a unit increase in the log-risk surface from one location to another implies the doubling of risk.

Now, for the inclusion of prognostic factor effects, a logit function can be used to link factor effects and an additive spatial effect to the probability of resistance *p*(*s*),

(1) $$log\left\{\frac{p(s,x)}{\text{1}-p(s,x)}\right\}=\beta x+g(s),$$

where *x *is the vector of individual/local prognostic factors, *β *are the effects and *g*(*s*) is a smooth (unknown) function of *s*.

If the risk is constant then *g*(*s*) = 0 and model (Equation I) becomes the usual logistic regression model [22]. Hence, model (Equation I) is the usual logistic regression model extended by an additive component *g*(*s*), so the interpretation of factor effects and the spatial effect are made accordingly.

Then, our model is as follows:

$$log\left\{\frac{p(s,x)}{\text{1}-p(s,x)}\right\}=\beta_{0}\;+\beta_{1}\;\text{D\_DDDA}+\;\beta_{2}{\text{I}}_{\text{1}}+\;\beta_{3}{\text{I}}_{\text{2}}+g(s)$$

where *β*_0_: regression model intercept; *β*_1_, *β*_2_, *and β*_3_: covariates effects; D_DDDA: antimicrobial usage mean density; I_1 _= 1 if T_1_< D_DDDA <T_2 _and I_1 _= 0 otherwise; I_2 _= 1 if D_DDDA > T_2_; and I_2 _= 0 otherwise. *E. coli *resistance change points (T_1 _and T_2_)_were identified after performing an initial analysis on the data by fitting an ordinary logistic regression model without the spatial term but allowing for an additive (smooth) effect of D_DDDA upon the risk of *E. coli *ciprofloxacin resistance.

## Results

**Figure 4:**
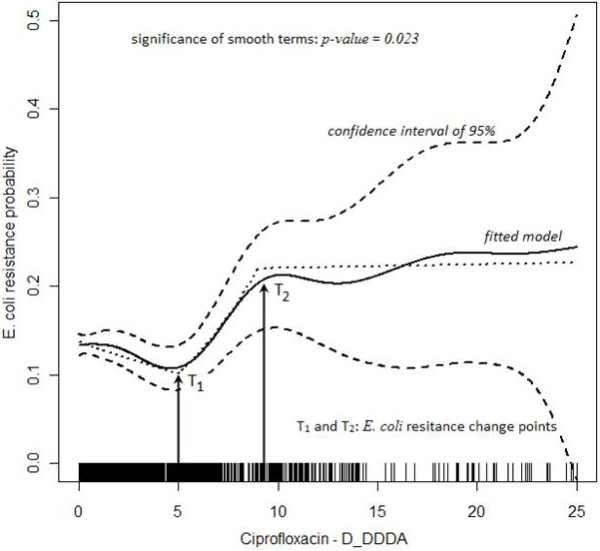
***E. coli *ciprofloxacin resistance probability VS. ciprofloxacin D_DDDA**.

**Figure 5:**
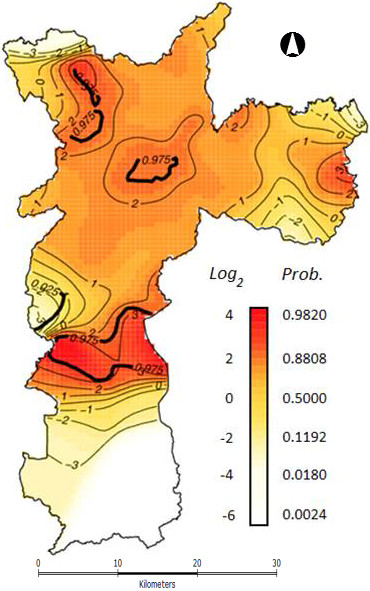
**São Paulo city estimated residual (log) risk map showing hot-spots of ciprofloxacin**.

The model has shown two major results: i) the probability of ciprofloxacin resistance in community UTI *E. coli *isolates was associated to population consumption level between 5 and 9 DDDs/1,000 inhabitants-day (T_1 _= 5 and T_2 _= 9), as an accumulated mean for the previous three months of the occurrence (D_DDDA) (Figure 4); ii) there was a significant global spatial variation in the residual risk of resistance (*p-value *= 0.002) after allowing for D_DDDA with hot-spots clustered in the city (Figure 5). These areas are delimited by 2.5% and 97.5% contours resulting approximate 95% tolerance contours identifying areas of even lower and higher risks, respectively, of *E. coli *ciprofloxacin resistance than those expected from the fitted model shown in Figure 4. Thus, the CIP consumption mean for the entire study area in year 2002 was 0.6 DDD/1,000 inhabitants-day and the level of D_DDDA which triggered the occurrence of resistance in this study was established between 5 and 9 DDDs of ciprofloxacin/1,000 inhabitants-day. Additionally, ciprofloxacin D_DDDA was found to be related to the clusters (Figure 5). However, these elevated risk areas of clustered ciprofloxacin *E. coli *resistance could have an enhanced risk of presenting other population covariates associated to its formations.

Additionally a sensitivity analysis was performed and although correlation between spatial effects and regression coefficients for spatially varying covariates may be a general concern, it did not prove relevant for the present data analysis. A generalized additive model allowing for a smooth function of D_DDDA in the linear predictor replacing the change point model together with a smooth spatial effect was also applied (solid line shown in Figure 4) and results maintained their significance at 5% level. Furthermore, the presented model has a comparable goodness of fit with the GAM model, thus being chosen for the present study due to its clearer interpretation.

## Discussion and Conclusions

Different epidemiological determinants may favor the emergence or establishment of specific resistances in given environments [5,23-25], in particular after shown that resistance lies within the human commensal bacterial species, which were once considered relatively harmless residents of the human microbiome [26]. It also seems reasonable to speculate that a human-driven increase in antibiotic concentrations of a given ecosystems, such as a city, may influence both antibiotic resistance and the microbial population dynamics [27]. As previously noted [3], a population could experience indirect effects of antimicrobial use, such as an enhanced risk for resistance acquisition, because of antimicrobial usage by others in the population. Nevertheless, it is still unknown how these different environmental and individual determinants are distributed over space and time and their possible influences on a resistance emergence or clonal spread. It has been shown by time-series analysis that antimicrobial usage in a restricted and contained environment, such as a hospital, is temporally linked to the emergence of bacterial resistance [9,10]. However, no correlation of antimicrobial population usage and bacterial resistance in the community had been established before the present study.

The present model used selling points as the basis for influence zone determination and this concept was supported by the premise that pharmacy consumption patterns, either by direct buy or delivery, originates from a zone closer to the subject`s residence. Nevertheless, authors do acknowledge that potential biases may exist in this concept, since consumption patterns may also be related to a subject`s habit of using pharmacies elsewhere in the city and that an overestimation of CIP usage may occur in commercial areas of a city. However, based on this model, it was found that the level of D_DDDA which triggered the occurrence of resistance in this study was established between 5 and 9 DDDs of CIP/1,000 inhabitants-day and that CIP D_DDDA was found to be related to the clusters. It means that in a population of 1,000 inhabitants in a given day, if a mean of 5 to 9 DDDs of ciprofloxacin had been consumed over the past three months, *E. coli *resistance would occur in subjects of the same geographical area (not necessarily the same individuals taking the medication). However, the model could not rule out other population covariates which might be associated to the elevated risk areas of clustered *E. coli *CIP resistance. Nevertheless, the model allows for the inclusion of many different covariates, or risk factors, to be investigated, as long as they can be geo-referenced, once other population covariates could be associated to the CIP resistant *E. coli *clusters.

We do envisage a combined approach to explore associated risk factors, since molecular epidemiology approaches have given insights into the area. Recently, the occurrence of an ecological phenomenon has been demonstrated [24] by a single clonal group accounting for nearly half of community-acquired urinary tract infections in women caused by *E. coli *with resistance to trimethoprim-sulfamethoxazole in three geographically diverse communities. In a different study, a geographical information system and a Bernoulli regression model were applied to detect clusters of higher risk of acquisition of *S. aureus *soft tissue abscesses [28]. Also, there have been strategies used with spatial scan statistics to identifying clusters of samples and to detecting areas with significantly high or low sampling rates of a national antimicrobial resistance monitoring program [29]. Jones et al. [7] applied a spatial analysis methodology and found more than half of emerging infectious diseases events to be caused by bacteria or rickettsia, with a large number of drug-resistance and significant correlation with socio-economic, environmental and ecological factors. There have been other significant efforts to understanding resistance dynamics and transmission with different approaches. It should be noticed that at least two other strategies [30,31] were based on bacterial surveillance data: McCormick et al [30] have shown patterns of geographic variation explained by the differences between the proportions of resistance in specific *S. pneumoniae *serotypes. Stelling et al [31] used an electronic laboratory data system (WHONET) and a space-time permutation scan statistic for generating a semi-automated detection of disease outbreak with *Shigella *spp [31]. Both latter approaches seem to be complimentary to the present one. Furthermore, all these approaches, including the present one, adopt a population-level perspective to elucidate the infection emergence problem and risk factors associated, in particular the resistance issue.

On the one hand individual-level studies do not provide the opportunity to observe the ecological phenomena, or indirect effects of treatment on resistance [3,32]. On the other, although avoiding the statistical non-independence issue between samples, population-level studies have not been able to provide a replicable framework on which one may test the hypothesis of the indirect effects of treatment on resistance.

The present model has been applied on a longitudinal observational study, and as such, conclusions are limited to the environment used, even considering the elevated sample size and the goodness of fit of model. As a cluster-randomized study of antimicrobial resistance, we applied the ratio of resistant to susceptible as an endpoint. The spatial clustering of antimicrobial resistance in this population points to an ecological effect. Nevertheless, the adopted framework allows for the inclusion of any number of temporal and spatial variables. In particular, for the study of resistance emergence, it is possible to fit in the model virtually any antimicrobial-microorganism combination, in pairs or in groups, if the objective is to observe class effects. We anticipate our spatial-temporal modelling strategy embedded into a geographic information system [33] analytical framework to be the start-up point for validating, replicating, and understanding antimicrobial resistance in community and for public health interventions.

## Competing interests

The authors declare that they have no competing interests.

## Authors' contributions

CRVK designed, collected, performed part of the analysis (Kernel), reviewed the data, and wrote the report. ECGC collected, standardized the database, critically reviewed the experiment design, and performed part of the analysis (Kernel, Voronnoi, Theme Maps, Model). SES and PJRJ reviewed the experiment, proposed the model, and performed the analysis (Model, Voronnoi). ACCP critically reviewed the experiment and helped interpreting and discussing the microbiological data. TCB proposed the analytical model. AMVM coordinated, designed supervised collection of data, reviewed the data, performed part of the analysis, and wrote the report. All authors read, approved and contributed equally the final manuscript.
